# *rac*-Dimethyl 5-oxo-2-[(2,4,4-trimethyl­pentan-2-yl)amino]-4,5-dihydro­pyrano[3,2-*c*]chromene-3,4-dicarboxyl­ate

**DOI:** 10.1107/S1600536812027596

**Published:** 2012-06-23

**Authors:** S. Antony Inglebert, Yuvaraj Arun, K. Sethusankar, Paramasivam T. Perumal

**Affiliations:** a Sri Ram Engineering College, Chennai 602 024, India; b Organic Chemistry Division, Central Leather Research Institute, Adyar, Chennai 600 020, India; c Department of Physics, RKM Vivekananda College (Autonomous), Chennai 600 004, India

## Abstract

The title compound, C_24_H_29_NO_7_, is asymmetric with a chiral centre located in the pyran ring and crystallizes as a racemate. The coumarin ring system and the fused pyran ring make a dihedral angle of 10.46 (8)°. A short intra­molecular N—H⋯O hydrogen bond between the amino group and the vicinal carbonyl group generates an *S*(6) ring. Inter­molecular C—H⋯O inter­actions contribute to the stability of the crystal structure.

## Related literature

For the biological activity of pyran­ocoumarin compounds, see: Kawaii *et al.* (2001 ▶); Goel *et al.* (1997 ▶); Xu *et al.* (2006 ▶). For a similar compound, see: Inglebert *et al.* (2011 ▶). For bond-angle distortions, see: Chinnakali *et al.* (1998 ▶); Kumar *et al.* (1997 ▶). For graph-set notation, see: Bernstein *et al.* (1995 ▶).
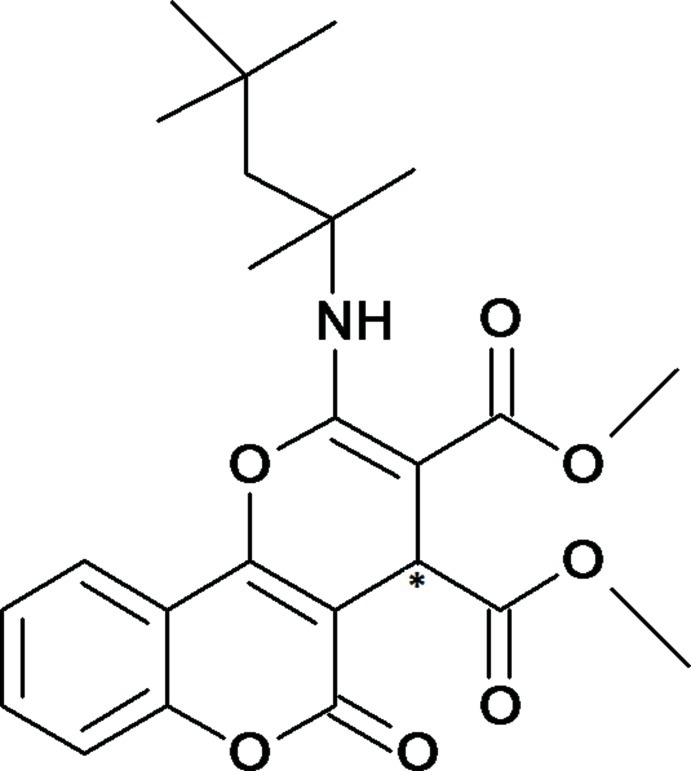


## Experimental

### 

#### Crystal data

C_24_H_29_NO_7_
*M* *_r_* = 443.48
Monoclinic, 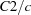

*a* = 22.0329 (17) Å
*b* = 11.8675 (8) Å
*c* = 18.4861 (14) Å
β = 107.946 (4)°
*V* = 4598.5 (6) Å^3^
*Z* = 8
Mo *K*α radiation
μ = 0.09 mm^−1^
*T* = 293 K
0.35 × 0.25 × 0.20 mm

#### Data collection

Bruker Kappa APEXII CCD diffractometer
Absorption correction: multi-scan (*SADABS*; Bruker, 2008 ▶) *T*_min_ = 0.972, *T*_max_ = 0.981
17657 measured reflections
3384 independent reflections
2623 reflections with *I* > 2σ(*I*)
*R*_int_ = 0.035
θ_max_ = 23.5°

#### Refinement

*R*[*F* ^2^ > 2σ(*F* ^2^)] = 0.044
*wR*(*F* ^2^) = 0.130
*S* = 1.01
3384 reflections
296 parameters
H-atom parameters constrained
Δρ_max_ = 0.24 e Å^−3^
Δρ_min_ = −0.24 e Å^−3^

### 

Data collection: *APEX2* (Bruker, 2008 ▶); cell refinement: *SAINT* (Bruker, 2008 ▶); data reduction: *SAINT*; program(s) used to solve structure: *SHELXS97* (Sheldrick, 2008 ▶); program(s) used to refine structure: *SHELXL97* (Sheldrick, 2008 ▶); molecular graphics: *ORTEP-3* (Farrugia, 1997 ▶); software used to prepare material for publication: *SHELXL97* and *PLATON* (Spek, 2009 ▶).

## Supplementary Material

Crystal structure: contains datablock(s) global, I. DOI: 10.1107/S1600536812027596/fy2059sup1.cif

Structure factors: contains datablock(s) I. DOI: 10.1107/S1600536812027596/fy2059Isup2.hkl

Supplementary material file. DOI: 10.1107/S1600536812027596/fy2059Isup3.cml

Additional supplementary materials:  crystallographic information; 3D view; checkCIF report

## Figures and Tables

**Table 1:** Hydrogen-bond geometry (Å, °)

*D*—H⋯*A*	*D*—H	H⋯*A*	*D*⋯*A*	*D*—H⋯*A*
C19—H19*C*⋯O2^i^	0.96	2.58	3.458 (3)	153
C23—H23*A*⋯O6^ii^	0.96	2.54	3.255 (4)	131
C23—H23*B*⋯O1^iii^	0.96	2.55	3.509 (4)	174
N1—H1⋯O6	0.86	2.01	2.660 (2)	131

Symmetry codes: (i) 
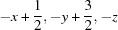
; (ii) 
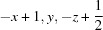
; (iii) 
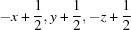
.

**Fig. 1.**
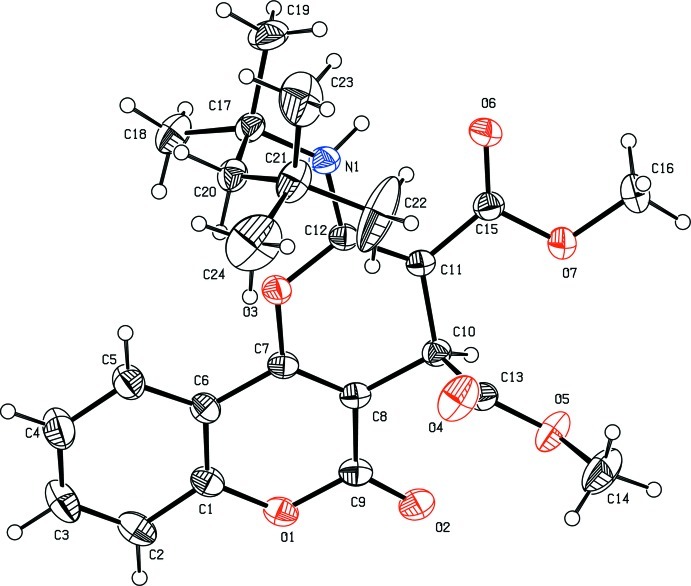
The asymmetric unit of the title compound, showing 30% probability displacement ellipsoids. H atoms are present as small spheres of arbitary radius.

**Fig. 2.**
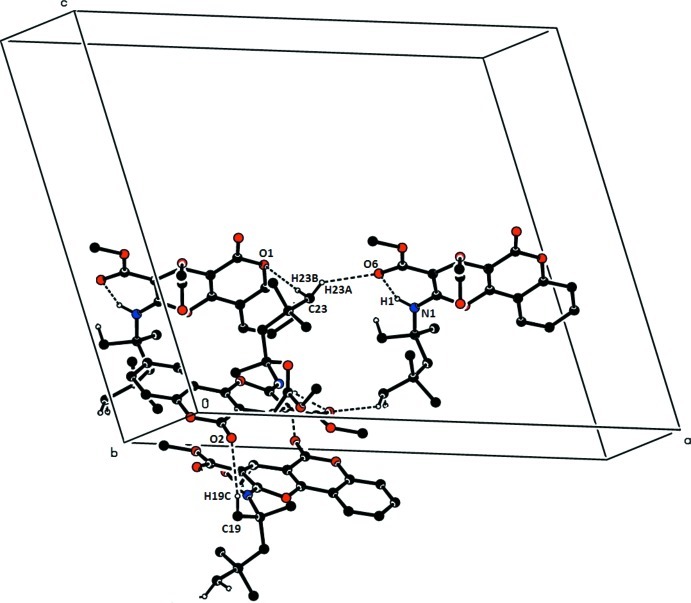
Crystal packing of the title compound, viewed along the b axis. Intermolecular C—H···O and intramolecular N—H···O interactions are shown as dashed lines. For the clarity, H atoms not involved in these interactions have been omited.

## supplementary crystallographic information

### Comment

Coumarins are natural or synthetic compounds used as pharmaceuticals and herbicides. They exhibit fluorescent properties due to the presence of the benzopyrone moiety. Pyranocoumarin and its derivatives show strong activity against cancer cell lines (Kawaii *et al.*, 2001). Some naturally occurring pyranocoumarins show antiulcer activity, anti-hepatitis B virus activity, cytotoxic activities and anti-TB activity (Goel *et al.*, 1997 and Xu *et al.*, 2006). Outside the biological applications of coumarin and its derivatives, there are also applications as cosmetics, optical brightening agents and laser dyes.

Fused benzene and pyranoid rings form the benzopyran system, which can be described as planar, with the dihedral angle between the best planes of the rings being 2.07 (11)°. The coumarin ring system, consisting of atoms C1–C6, C7–C9 and O1 and O2 is almost planar with maximum deviation from the mean plane of 0.033 (2) Å for C8. The coumarin ring system (O1/C1–C9) makes a dihedral angle of 10.46 (8)° with pyran ring (O3/C7–C12). The coumarin ring system and pyran ring make the dihedral angles of 77.56 (10)°, 17.74 (7)°, 87.03 (10)° and 9.74 (6)° with the two methyl carboxylates (C13/O4/O5/C14) and (C15/O6/O7/C16), respectively. The methyl carboxylates are almost perpendicular to each other because the dihedral angle between them is 88.84 (15)°.

In the benzopyran ring, the bond distances of O1–C9 and C9–C8 are 1.372 (3) Å and 1.448 (3) Å, respectively, indicating that the electrons are delocalized in the ring with the carbonyl group acting as an electron-withdrawing group. This is corroborated by the fact that the benzopyran ring is planar. The title structure exhibits the structural similarities with our previously reported structure (Inglebert *et al.*, 2011). As observed in other coumarin derivatives, the C5–C6 and C7–C8 bonds in the coumarin moiety show double-bond character and steric interactions cause the widening of angles C8–C9–O2 (125.0 (2)°) and C7–C6–C5 (125.24 (19)°), and the narrowing of angles O1–C9–O2 (117.22 (18)°) and O1–C1–C2 (116.82 (19)°) from 120° (Chinnakali *et al.*, 1998; Kumar *et al.*, 1997).

The carbonyl oxygen atom O6 acts as a bifurcated acceptor, accepting both the intramoleclar N1—H1···O6 and the intermolecular C23—H23A···O6 hydrogen bonds. The intramolecular bond generates an *S*(6) ring motif (Bernstein *et al.*, 1995). The crystal packing is stabilized by intermolecular C—H···O interactions (Table 1).

### Experimental

To a magnetically stirred solution of 4-hydroxy coumarin (0.162 g, 1.0 mmol) and dimethyl acetylenedicarboxylate (0.142 g, 1.0 mmol) in CH_3_CN (10 ml) was added a solution of 1,1,3,3-tetra methylbutyl isocynaide (0.139 g, 1.0 mmol) at room temperature over 5 min. The mixture was then stirred for 24 h. After completion of the reaction, the solvent was removed under vacuum and the solid residue was washed with n-hexane and crystallized from CH_2_Cl_2_ – *n*-hexane (1/2) to give the product as white crystals (0.368 g, 83%).

### Refinement

The H atoms bound to the C and N atoms were placed geometrically and treated as riding atoms, with d(N—H) = 0.86 Å and *U*_iso_(H) = 1.2*U*_eq_(N) for the amino group, with d(C—H) = 0.93 Å and *U*_iso_(H) = 1.2*U*_eq_(C) for aromatic, d(C—H) = 0.97 Å and *U*_iso_(H) = 1.2*U*_eq_(C) for methylene and d(C—H) = 0.96 Å and *U*_iso_(H) = 1.5*U*_eq_(C) for methyl groups. The rotation angles for methyl groups were optimised by least squares.

### Crystal data

**Table:**

C_24_H_29_NO_7_	*F*(000) = 1888
*M**_r_* = 443.48	*D*_x_ = 1.281 Mg m^−^^3^
Monoclinic, *C*2/*c*	Mo *K*α radiation, λ = 0.71073 Å
Hall symbol: -C 2yc	Cell parameters from 3384 reflections
*a* = 22.0329 (17) Å	θ = 2.1–23.5°
*b* = 11.8675 (8) Å	µ = 0.09 mm^−^^1^
*c* = 18.4861 (14) Å	*T* = 293 K
β = 107.946 (4)°	Block, colourless
*V* = 4598.5 (6) Å^3^	0.35 × 0.25 × 0.20 mm
*Z* = 8	

### Data collection

**Table:**

Bruker Kappa APEXII CCD diffractometer	3384 independent reflections
Radiation source: fine-focus sealed tube	2623 reflections with *I* > 2σ(*I*)
Graphite monochromator	*R*_int_ = 0.035
ω and φ scan	θ_max_ = 23.5°, θ_min_ = 2.1°
Absorption correction: multi-scan (*SADABS*; Bruker, 2008)	*h* = −24→24
*T*_min_ = 0.972, *T*_max_ = 0.981	*k* = −13→13
17657 measured reflections	*l* = −20→20

### Refinement

**Table:**

Refinement on *F*^2^	Primary atom site location: structure-invariant direct methods
Least-squares matrix: full	Secondary atom site location: difference Fourier map
*R*[*F*^2^ > 2σ(*F*^2^)] = 0.044	Hydrogen site location: inferred from neighbouring sites
*wR*(*F*^2^) = 0.130	H-atom parameters constrained
*S* = 1.01	*w* = 1/[σ^2^(*F*_o_^2^) + (0.0745*P*)^2^ + 2.5421*P*] where *P* = (*F*_o_^2^ + 2*F*_c_^2^)/3
3384 reflections	(Δ/σ)_max_ < 0.001
296 parameters	Δρ_max_ = 0.24 e Å^−^^3^
0 restraints	Δρ_min_ = −0.24 e Å^−^^3^

### Special details

**Table:**

Geometry. All e.s.d.'s (except the e.s.d. in the dihedral angle between two l.s. planes) are estimated using the full covariance matrix. The cell e.s.d.'s are taken into account individually in the estimation of e.s.d.'s in distances, angles and torsion angles; correlations between e.s.d.'s in cell parameters are only used when they are defined by crystal symmetry. An approximate (isotropic) treatment of cell e.s.d.'s is used for estimating e.s.d.'s involving l.s. planes.
Refinement. Refinement of *F*^2^ against ALL reflections. The weighted *R*-factor *wR* and goodness of fit *S* are based on *F*^2^, conventional *R*-factors *R* are based on *F*, with *F* set to zero for negative *F*^2^. The threshold expression of *F*^2^ > σ(*F*^2^) is used only for calculating *R*-factors(gt) *etc*. and is not relevant to the choice of reflections for refinement. *R*-factors based on *F*^2^ are statistically about twice as large as those based on *F*, and *R*- factors based on ALL data will be even larger.

### Fractional atomic coordinates and isotropic or equivalent isotropic displacement parameters (Å^2^)

**Table:**

	*x*	*y*	*z*	*U*_iso_*/*U*_eq_	
C1	0.07781 (10)	0.65241 (18)	0.08539 (12)	0.0434 (5)	
C2	0.01923 (11)	0.6706 (2)	0.09593 (14)	0.0580 (6)	
H2	−0.0163	0.6291	0.0690	0.070*	
C3	0.01469 (13)	0.7514 (3)	0.14700 (16)	0.0707 (8)	
H3	−0.0245	0.7649	0.1545	0.085*	
C4	0.06739 (12)	0.8128 (2)	0.18737 (15)	0.0666 (7)	
H4	0.0634	0.8669	0.2220	0.080*	
C5	0.12543 (11)	0.7948 (2)	0.17689 (13)	0.0538 (6)	
H5	0.1607	0.8367	0.2042	0.065*	
C6	0.13161 (10)	0.71310 (18)	0.12499 (12)	0.0411 (5)	
C7	0.18985 (9)	0.68570 (17)	0.10922 (11)	0.0362 (5)	
C8	0.19380 (9)	0.60197 (16)	0.06255 (11)	0.0356 (5)	
C9	0.13680 (10)	0.54036 (18)	0.02177 (12)	0.0408 (5)	
C10	0.25577 (9)	0.56956 (16)	0.05062 (11)	0.0359 (5)	
H10	0.2486	0.5539	−0.0034	0.043*	
C11	0.30259 (9)	0.66519 (16)	0.07534 (10)	0.0347 (5)	
C12	0.29355 (9)	0.74938 (16)	0.12167 (11)	0.0348 (5)	
C13	0.28234 (10)	0.46404 (17)	0.09713 (13)	0.0413 (5)	
C14	0.31163 (16)	0.2746 (2)	0.09316 (18)	0.0842 (9)	
H14A	0.3533	0.2905	0.1276	0.126*	
H14B	0.3150	0.2190	0.0569	0.126*	
H14C	0.2847	0.2465	0.1212	0.126*	
C15	0.36146 (10)	0.66180 (18)	0.05798 (11)	0.0412 (5)	
C16	0.42747 (14)	0.5498 (2)	0.0079 (2)	0.0802 (9)	
H16A	0.4391	0.6164	−0.0141	0.120*	
H16B	0.4244	0.4873	−0.0260	0.120*	
H16C	0.4594	0.5341	0.0555	0.120*	
C17	0.33589 (10)	0.91740 (17)	0.20939 (12)	0.0419 (5)	
C18	0.27751 (13)	0.99465 (19)	0.18546 (14)	0.0598 (7)	
H18A	0.2732	1.0257	0.1362	0.090*	
H18B	0.2827	1.0546	0.2217	0.090*	
H18C	0.2400	0.9521	0.1835	0.090*	
C19	0.39446 (13)	0.9891 (2)	0.21481 (15)	0.0643 (7)	
H19A	0.4314	0.9415	0.2250	0.096*	
H19B	0.4005	1.0429	0.2552	0.096*	
H19C	0.3885	1.0282	0.1676	0.096*	
C20	0.33782 (10)	0.85921 (18)	0.28459 (11)	0.0442 (5)	
H20A	0.3367	0.9190	0.3199	0.053*	
H20B	0.2979	0.8186	0.2746	0.053*	
C21	0.39048 (12)	0.7775 (2)	0.32812 (13)	0.0598 (7)	
C22	0.4003 (2)	0.6793 (3)	0.28107 (18)	0.1349 (19)	
H22A	0.3602	0.6425	0.2579	0.202*	
H22B	0.4296	0.6268	0.3132	0.202*	
H22C	0.4173	0.7060	0.2422	0.202*	
C23	0.45323 (14)	0.8378 (3)	0.36641 (19)	0.1006 (11)	
H23A	0.4809	0.7889	0.4035	0.151*	
H23B	0.4451	0.9049	0.3909	0.151*	
H23C	0.4733	0.8577	0.3289	0.151*	
C24	0.3680 (2)	0.7316 (3)	0.39292 (19)	0.1155 (13)	
H24A	0.3293	0.6894	0.3721	0.173*	
H24B	0.3602	0.7932	0.4225	0.173*	
H24C	0.4003	0.6834	0.4247	0.173*	
N1	0.33318 (8)	0.83453 (14)	0.14823 (9)	0.0427 (4)	
H1	0.3621	0.8432	0.1264	0.051*	
O1	0.08075 (6)	0.56951 (12)	0.03460 (8)	0.0464 (4)	
O2	0.13476 (7)	0.46490 (13)	−0.02235 (9)	0.0550 (4)	
O3	0.24034 (6)	0.75349 (12)	0.14530 (8)	0.0416 (4)	
O4	0.29947 (10)	0.46127 (14)	0.16463 (10)	0.0717 (6)	
O5	0.28442 (8)	0.37646 (12)	0.05382 (9)	0.0620 (5)	
O6	0.40352 (7)	0.73256 (13)	0.07379 (9)	0.0530 (4)	
O7	0.36707 (8)	0.56715 (13)	0.02049 (10)	0.0614 (5)	

### Atomic displacement parameters (Å^2^)

**Table:**

	*U*^11^	*U*^22^	*U*^33^	*U*^12^	*U*^13^	*U*^23^
C1	0.0370 (13)	0.0505 (13)	0.0409 (12)	−0.0018 (10)	0.0096 (9)	0.0054 (11)
C2	0.0359 (13)	0.0777 (17)	0.0587 (15)	−0.0055 (12)	0.0120 (11)	0.0052 (14)
C3	0.0436 (15)	0.099 (2)	0.0752 (18)	0.0093 (15)	0.0270 (13)	−0.0021 (17)
C4	0.0557 (17)	0.0817 (18)	0.0679 (17)	0.0057 (14)	0.0272 (14)	−0.0140 (14)
C5	0.0436 (14)	0.0648 (15)	0.0543 (14)	−0.0019 (12)	0.0168 (11)	−0.0102 (12)
C6	0.0349 (12)	0.0460 (12)	0.0417 (12)	0.0010 (10)	0.0108 (9)	0.0014 (10)
C7	0.0315 (11)	0.0392 (11)	0.0345 (11)	−0.0039 (9)	0.0052 (8)	0.0002 (9)
C8	0.0345 (12)	0.0339 (11)	0.0355 (11)	−0.0043 (9)	0.0067 (9)	0.0021 (9)
C9	0.0393 (13)	0.0406 (12)	0.0399 (12)	−0.0048 (10)	0.0086 (9)	0.0051 (10)
C10	0.0366 (11)	0.0358 (11)	0.0345 (11)	−0.0039 (9)	0.0098 (9)	−0.0032 (9)
C11	0.0337 (11)	0.0332 (11)	0.0358 (10)	−0.0038 (9)	0.0087 (9)	−0.0006 (9)
C12	0.0318 (11)	0.0360 (11)	0.0346 (11)	−0.0026 (9)	0.0075 (9)	0.0006 (9)
C13	0.0381 (12)	0.0374 (12)	0.0474 (14)	−0.0037 (9)	0.0117 (10)	−0.0030 (10)
C14	0.096 (2)	0.0413 (14)	0.102 (2)	0.0201 (15)	0.0102 (18)	−0.0025 (14)
C15	0.0416 (13)	0.0395 (12)	0.0424 (12)	−0.0045 (11)	0.0128 (10)	−0.0022 (10)
C16	0.0652 (18)	0.0710 (18)	0.123 (3)	−0.0001 (15)	0.0564 (18)	−0.0253 (17)
C17	0.0446 (13)	0.0368 (11)	0.0428 (12)	−0.0036 (10)	0.0111 (10)	−0.0089 (9)
C18	0.0705 (17)	0.0421 (13)	0.0578 (14)	0.0103 (12)	0.0064 (12)	−0.0015 (11)
C19	0.0749 (18)	0.0552 (15)	0.0645 (15)	−0.0293 (13)	0.0243 (14)	−0.0208 (12)
C20	0.0428 (13)	0.0444 (12)	0.0423 (12)	0.0008 (10)	0.0086 (10)	−0.0074 (10)
C21	0.0651 (17)	0.0523 (14)	0.0490 (14)	0.0163 (12)	−0.0016 (12)	−0.0029 (11)
C22	0.192 (4)	0.088 (2)	0.079 (2)	0.089 (3)	−0.025 (2)	−0.0216 (19)
C23	0.060 (2)	0.117 (3)	0.101 (2)	0.0290 (18)	−0.0102 (17)	−0.010 (2)
C24	0.153 (4)	0.099 (3)	0.079 (2)	0.022 (2)	0.014 (2)	0.038 (2)
N1	0.0449 (11)	0.0427 (10)	0.0434 (10)	−0.0124 (9)	0.0178 (8)	−0.0107 (8)
O1	0.0352 (9)	0.0525 (9)	0.0489 (9)	−0.0083 (7)	0.0090 (7)	−0.0021 (7)
O2	0.0519 (10)	0.0524 (10)	0.0580 (10)	−0.0149 (8)	0.0129 (8)	−0.0181 (8)
O3	0.0341 (8)	0.0466 (8)	0.0442 (8)	−0.0070 (7)	0.0125 (6)	−0.0120 (7)
O4	0.1079 (15)	0.0542 (10)	0.0471 (11)	0.0141 (10)	0.0153 (10)	0.0083 (8)
O5	0.0769 (12)	0.0363 (9)	0.0641 (10)	0.0097 (8)	0.0087 (9)	−0.0089 (8)
O6	0.0446 (9)	0.0528 (9)	0.0674 (10)	−0.0145 (8)	0.0259 (8)	−0.0139 (8)
O7	0.0536 (10)	0.0524 (10)	0.0898 (12)	−0.0100 (8)	0.0391 (9)	−0.0266 (9)

### Geometric parameters (Å, º)

**Table:**

C1—O1	1.375 (3)	C15—O7	1.345 (3)
C1—C2	1.380 (3)	C16—O7	1.434 (3)
C1—C6	1.388 (3)	C16—H16A	0.9600
C2—C3	1.370 (4)	C16—H16B	0.9600
C2—H2	0.9300	C16—H16C	0.9600
C3—C4	1.379 (4)	C17—N1	1.486 (3)
C3—H3	0.9300	C17—C19	1.523 (3)
C4—C5	1.368 (3)	C17—C18	1.530 (3)
C4—H4	0.9300	C17—C20	1.541 (3)
C5—C6	1.400 (3)	C18—H18A	0.9600
C5—H5	0.9300	C18—H18B	0.9600
C6—C7	1.438 (3)	C18—H18C	0.9600
C7—C8	1.336 (3)	C19—H19A	0.9600
C7—O3	1.368 (2)	C19—H19B	0.9600
C8—C9	1.448 (3)	C19—H19C	0.9600
C8—C10	1.499 (3)	C20—C21	1.535 (3)
C9—O2	1.203 (2)	C20—H20A	0.9700
C9—O1	1.372 (3)	C20—H20B	0.9700
C10—C11	1.507 (3)	C21—C22	1.510 (4)
C10—C13	1.530 (3)	C21—C23	1.524 (4)
C10—H10	0.9800	C21—C24	1.531 (4)
C11—C12	1.370 (3)	C22—H22A	0.9600
C11—C15	1.429 (3)	C22—H22B	0.9600
C12—N1	1.327 (2)	C22—H22C	0.9600
C12—O3	1.372 (2)	C23—H23A	0.9600
C13—O4	1.188 (3)	C23—H23B	0.9600
C13—O5	1.321 (2)	C23—H23C	0.9600
C14—O5	1.443 (3)	C24—H24A	0.9600
C14—H14A	0.9600	C24—H24B	0.9600
C14—H14B	0.9600	C24—H24C	0.9600
C14—H14C	0.9600	N1—H1	0.8600
C15—O6	1.217 (2)		
			
O1—C1—C2	116.82 (19)	H16B—C16—H16C	109.5
O1—C1—C6	121.28 (18)	N1—C17—C19	104.75 (17)
C2—C1—C6	121.9 (2)	N1—C17—C18	110.10 (17)
C3—C2—C1	118.5 (2)	C19—C17—C18	107.69 (19)
C3—C2—H2	120.8	N1—C17—C20	111.92 (16)
C1—C2—H2	120.8	C19—C17—C20	113.81 (18)
C2—C3—C4	121.0 (2)	C18—C17—C20	108.45 (18)
C2—C3—H3	119.5	C17—C18—H18A	109.5
C4—C3—H3	119.5	C17—C18—H18B	109.5
C5—C4—C3	120.6 (2)	H18A—C18—H18B	109.5
C5—C4—H4	119.7	C17—C18—H18C	109.5
C3—C4—H4	119.7	H18A—C18—H18C	109.5
C4—C5—C6	119.9 (2)	H18B—C18—H18C	109.5
C4—C5—H5	120.1	C17—C19—H19A	109.5
C6—C5—H5	120.1	C17—C19—H19B	109.5
C1—C6—C5	118.2 (2)	H19A—C19—H19B	109.5
C1—C6—C7	116.52 (19)	C17—C19—H19C	109.5
C5—C6—C7	125.24 (19)	H19A—C19—H19C	109.5
C8—C7—O3	123.17 (18)	H19B—C19—H19C	109.5
C8—C7—C6	122.59 (18)	C21—C20—C17	124.20 (19)
O3—C7—C6	114.24 (17)	C21—C20—H20A	106.3
C7—C8—C9	119.55 (19)	C17—C20—H20A	106.3
C7—C8—C10	121.96 (17)	C21—C20—H20B	106.3
C9—C8—C10	118.50 (17)	C17—C20—H20B	106.3
O2—C9—O1	117.22 (18)	H20A—C20—H20B	106.4
O2—C9—C8	125.0 (2)	C22—C21—C23	111.1 (3)
O1—C9—C8	117.73 (19)	C22—C21—C24	108.3 (3)
C8—C10—C11	109.38 (16)	C23—C21—C24	105.7 (3)
C8—C10—C13	109.61 (16)	C22—C21—C20	113.9 (2)
C11—C10—C13	109.78 (16)	C23—C21—C20	112.2 (2)
C8—C10—H10	109.4	C24—C21—C20	105.1 (2)
C11—C10—H10	109.4	C21—C22—H22A	109.5
C13—C10—H10	109.4	C21—C22—H22B	109.5
C12—C11—C15	118.54 (17)	H22A—C22—H22B	109.5
C12—C11—C10	121.39 (17)	C21—C22—H22C	109.5
C15—C11—C10	119.64 (17)	H22A—C22—H22C	109.5
N1—C12—C11	125.51 (18)	H22B—C22—H22C	109.5
N1—C12—O3	112.61 (16)	C21—C23—H23A	109.5
C11—C12—O3	121.87 (17)	C21—C23—H23B	109.5
O4—C13—O5	123.8 (2)	H23A—C23—H23B	109.5
O4—C13—C10	123.77 (19)	C21—C23—H23C	109.5
O5—C13—C10	112.47 (18)	H23A—C23—H23C	109.5
O5—C14—H14A	109.5	H23B—C23—H23C	109.5
O5—C14—H14B	109.5	C21—C24—H24A	109.5
H14A—C14—H14B	109.5	C21—C24—H24B	109.5
O5—C14—H14C	109.5	H24A—C24—H24B	109.5
H14A—C14—H14C	109.5	C21—C24—H24C	109.5
H14B—C14—H14C	109.5	H24A—C24—H24C	109.5
O6—C15—O7	121.03 (19)	H24B—C24—H24C	109.5
O6—C15—C11	127.07 (19)	C12—N1—C17	130.79 (17)
O7—C15—C11	111.89 (18)	C12—N1—H1	114.6
O7—C16—H16A	109.5	C17—N1—H1	114.6
O7—C16—H16B	109.5	C9—O1—C1	122.20 (16)
H16A—C16—H16B	109.5	C7—O3—C12	118.03 (15)
O7—C16—H16C	109.5	C13—O5—C14	116.13 (19)
H16A—C16—H16C	109.5	C15—O7—C16	116.12 (18)
			
O1—C1—C2—C3	179.0 (2)	C15—C11—C12—O3	−175.20 (17)
C6—C1—C2—C3	0.1 (3)	C10—C11—C12—O3	−2.7 (3)
C1—C2—C3—C4	−0.4 (4)	C8—C10—C13—O4	−65.5 (3)
C2—C3—C4—C5	0.4 (4)	C11—C10—C13—O4	54.7 (3)
C3—C4—C5—C6	−0.2 (4)	C8—C10—C13—O5	114.86 (19)
O1—C1—C6—C5	−178.78 (19)	C11—C10—C13—O5	−124.98 (18)
C2—C1—C6—C5	0.0 (3)	C12—C11—C15—O6	−7.8 (3)
O1—C1—C6—C7	0.8 (3)	C10—C11—C15—O6	179.6 (2)
C2—C1—C6—C7	179.6 (2)	C12—C11—C15—O7	171.88 (18)
C4—C5—C6—C1	0.0 (3)	C10—C11—C15—O7	−0.7 (3)
C4—C5—C6—C7	−179.6 (2)	N1—C17—C20—C21	60.1 (3)
C1—C6—C7—C8	−3.9 (3)	C19—C17—C20—C21	−58.4 (3)
C5—C6—C7—C8	175.7 (2)	C18—C17—C20—C21	−178.2 (2)
C1—C6—C7—O3	175.31 (17)	C17—C20—C21—C22	−55.2 (4)
C5—C6—C7—O3	−5.1 (3)	C17—C20—C21—C23	72.1 (3)
O3—C7—C8—C9	−174.87 (17)	C17—C20—C21—C24	−173.5 (2)
C6—C7—C8—C9	4.3 (3)	C11—C12—N1—C17	−166.6 (2)
O3—C7—C8—C10	4.9 (3)	O3—C12—N1—C17	13.7 (3)
C6—C7—C8—C10	−175.94 (18)	C19—C17—N1—C12	177.1 (2)
C7—C8—C9—O2	178.8 (2)	C18—C17—N1—C12	−67.3 (3)
C10—C8—C9—O2	−0.9 (3)	C20—C17—N1—C12	53.4 (3)
C7—C8—C9—O1	−1.6 (3)	O2—C9—O1—C1	178.21 (18)
C10—C8—C9—O1	178.63 (16)	C8—C9—O1—C1	−1.4 (3)
C7—C8—C10—C11	−19.0 (2)	C2—C1—O1—C9	−177.13 (18)
C9—C8—C10—C11	160.78 (17)	C6—C1—O1—C9	1.7 (3)
C7—C8—C10—C13	101.4 (2)	C8—C7—O3—C12	12.7 (3)
C9—C8—C10—C13	−78.8 (2)	C6—C7—O3—C12	−166.56 (16)
C8—C10—C11—C12	17.8 (2)	N1—C12—O3—C7	166.09 (16)
C13—C10—C11—C12	−102.5 (2)	C11—C12—O3—C7	−13.6 (3)
C8—C10—C11—C15	−169.79 (17)	O4—C13—O5—C14	−2.4 (3)
C13—C10—C11—C15	69.9 (2)	C10—C13—O5—C14	177.3 (2)
C15—C11—C12—N1	5.2 (3)	O6—C15—O7—C16	6.4 (3)
C10—C11—C12—N1	177.64 (18)	C11—C15—O7—C16	−173.3 (2)

### Hydrogen-bond geometry (Å, º)

**Table:**

*D*—H···*A*	*D*—H	H···*A*	*D*···*A*	*D*—H···*A*
C19—H19*C*···O2^i^	0.96	2.58	3.458 (3)	153
C23—H23*A*···O6^ii^	0.96	2.54	3.255 (4)	131
C23—H23*B*···O1^iii^	0.96	2.55	3.509 (4)	174
N1—H1···O6	0.86	2.01	2.660 (2)	131

Symmetry codes: (i) −*x*+1/2, −*y*+3/2, −*z*; (ii) −*x*+1, *y*, −*z*+1/2; (iii) −*x*+1/2, *y*+1/2, −*z*+1/2.
